# Biomass Flow and Scavengers Use of Carcasses after Re-Colonization of an Apex Predator

**DOI:** 10.1371/journal.pone.0077373

**Published:** 2013-10-23

**Authors:** Camilla Wikenros, Håkan Sand, Per Ahlqvist, Olof Liberg

**Affiliations:** Grimsö Wildlife Research Station, Department of Ecology, Swedish University of Agricultural Sciences, Riddarhyttan, Sweden; University of Alberta, Canada

## Abstract

**Background:**

Reestablishment of apex predators influences the availability and distribution of biomass for scavengers and can therefore be an important agent for structuring species communities. We studied how the re-colonization of the Scandinavian Peninsula by wolves (*Canis lupus*) affected the amount and temporal variation in use of moose (*Alces alces*) carcasses.

**Methodology/Principal Findings:**

We compared the availability of biomass from remains at wolf kills with those killed by hunters, vehicle collisions and natural death. Movement-triggered cameras monitored patterns of use on wolf kills and remains from hunter harvest by scavengers (n = 15 276) in relation to time of year, available carcass biomass, time since the death of the moose and presence of wolves. Remains from hunter harvest were the largest food source for scavengers both within wolf territories (57%) and in areas without wolves (81%). The total annual biomass available were similar in areas with (25 648 kg) and without (24 289 kg) wolves. Presence of wolves lowered the peak biomass available from hunter harvest in October (20%) and increased biomass available during December to August (38–324% per month). The probability of scavengers being present decreased faster with time at remains from hunter harvest compared to wolf kills and both the probability of being present and the number of visits by scavengers to wolf kills increased as the amount of biomass available on the carcass increased.

**Conclusions/Significance:**

Wolves reduced the seasonal variation of biomass from moose carcasses and most important increased it during spring. Scavengers also visited wolf kills most frequently during spring when most scavenging species have young, which may lead to an increase in survival and/or reproductive success of scavengers within wolf territories. This applies both for abundant scavenging species that were the most frequent visitors at wolf kills and threatened scavengers with lower visit frequency.

## Introduction

Scavenging is a common phenomenon among terrestrial vertebrates and almost all predators are scavengers to some extent [1]. Use of carcasses during periods of prey shortage, in stressful environmental situations, or as an alternative food resource may have substantial impacts on population dynamics and thus on the structure of scavenging communities [1]. In the temperate zone scavenging vertebrates mainly consume carcasses during the cold season [1] when other food sources become scarcer with the progress of winter [2]. For some scavenging species such as the red fox (*Vulpes vulpes*) and wolverine (*Gulo gulo*) reproductive success has been shown to increase with additional food during winter [3], [4].

Humans unintentionally provide food for scavengers through hunting and wildlife-vehicle collisions, but these sources of biomass have large temporal and spatial variations. Remains from hunter harvest are generally available only for a few months during the hunting season in autumn [5], and road density will affect the number of ungulate-vehicle collisions [6]. Thus, the temporal and spatial distribution of carcasses to scavengers will depend on the predominant cause of mortality in ungulate populations [1].

Removal, re-colonization, or reintroduction of apex predators in an ecosystem can have large effects on other species both through density- and behaviourally-mediated indirect interactions [7], [8], [9]. One example is the return of wolves (*Canis lupus*) to Yellowstone National Park, USA. Here, as elsewhere in northern ecosystems, winters are getting shorter as a result of climate change, which have resulted in fewer large ungulates dying of starvation [10]. The reintroduction of wolves have compensated for this decrease of winter carcasses by providing carcasses of prey with reduced seasonal and year-to-year variation compared to remains after hunter harvest [5] and winter starvation [10].

Scavengers may adjust their behaviour to locate carcass remains. The common raven (*Corvus corax*) is a species that commonly associates with wolves during winter as a foraging strategy to discover carcasses [11]. Also, the red fox seems to use wolves as guides to find kill remains by following their tracks in the snow [12]. However, predator kills are often consumed to a large extent by the predator itself [13], forcing scavengers to rely more on animals that have died from other causes than predation [1]. Scavenging kills by large predators is also a risky behaviour due to intra-guild predation [14]. Wolves often return to old kills [15] where they might surprise scavengers and kill them. For example, coyotes (*Canis latrans*) are known to scavenge on wolf-killed prey and the population in Yellowstone was reduced after the reintroduction of wolves [16], [17].

After a long period of absence, wolves have returned to the Scandinavian Peninsula through natural re-colonization [18]. In this ecosystem, moose is the main prey for wolves all year round [19], [20] and therefore they are a potential source of carcasses for scavengers. In addition, humans are a large provider of moose carcass remains to scavengers mainly through hunter harvest but also through vehicle collisions. We investigated how the re-colonization by wolves affected the availability of biomass from moose carcasses to scavengers and discuss the potential consequences for the scavenging guild. Specifically, we estimated the temporal variation in carcass biomass from wolf predation on moose over the year compared with biomass of moose from other causes of mortality: hunter harvest, vehicle collisions and natural death. We also compared the total amount of estimated available biomass from moose in areas with and without wolf predation. We then examined which scavenging species were found at carcasses after wolf-killed moose and at remains from hunter harvest and at what frequency in relation to time of year, available carcass biomass, time since the death of the moose, and presence of wolves. We hypothesized there would be an increase and a shift in the timing of available biomass for scavengers in areas when wolves were present [21].

### Study Site and Species

The study was conducted in the south-central part of the Scandinavian Peninsula (south-central Sweden and the adjacent eastern part of Norway, 59°–61°N, 12°–17°E, hereafter referred to as Scandinavia) in area consisting mainly of boreal forest. Most of the forests were managed by clear-cutting regeneration resulting in a mosaic of conifer stands in different age classes. The climate is characterized by continental climate with average temperatures of −5°C in January and 15°C in July [22]. The ground is usually snow covered between late November and early April with a mean snow depth of 20 cm in mid-January [23].

Wolves were extirpated from the study area and most of Scandinavia at the end of the 19th century and were functionally extinct by the 1960s [24]. They returned to the study area in the early 1980s through natural re-colonization and the first reproduction occurred in 1983 [18]. During the 1990s the wolf population increased both in numbers (29% average annual increase) and range [18]. By the winter of 2009/2010, the total population was estimated to be 252–291 wolves (28 packs and 21–24 pairs [25]) with the majority located in Sweden.

The Scandinavian moose population has been one of the most productive and most extensively harvested in the world since the 1960s [26]. About 100 000 individuals (25–30% of the pre-harvest moose population) were harvested annually in the beginning of the 21st century [26]. Winter densities of moose ranged between 0.6 and 2.5 moose/km^2^ [27], [28].

Large and medium-sized mammalian predators and potential scavengers in the area include the brown bear (*Ursus arctos*), Eurasian lynx (*Lynx lynx*), red fox, European badger (*Meles meles*), and European pine marten (*Martes martes*). According to a carcass utilization study in Poland [29], the most common avian scavengers were the Eurasian jay (*Garrulus glandarius*), raven, common buzzard (*Buteo buteo*), and white-tailed eagle (*Haliaeetus albicilla*). These species also occurred in our study area, although the white-tailed eagles is rare and is listed as near threatened [30].

## Methods

### Ethics Statement

All procedures including capture, handling and collaring of wolves [31] fulfilled ethical requirements and have been approved by the Swedish Animal Welfare Agency (Permit Number: C 281/6) and the Norwegian Experimental Animal Ethics Committee. The Swedish Animal Welfare Agency approved camera monitoring of scavenging species (Permit Number: C 51/9). Permission for camera monitoring of moose carcasses on both state-owned and privately owned land was obtained from the County Administrative Boards in Sweden (Dalarna (Permit Number: 211-14304-2006), Gävleborg (Permit Number: 211-1371-09), Värmland (Permit Number: 211-15846-06), Västmanland (Permit Number: 211-11827-06), and Örebro (Permit Number: 211-03990-2006)).

### Available Biomass for Scavengers

We estimated the amount of available biomass for scavenging species from moose carcasses killed by wolves, hunters harvest, vehicles, or from natural death. Available biomass (kg/month) was calculated for an average annual wolf territory of 900 km^2^ (95% MCP [32]) in areas with and without wolves. The calculations were based on data from four counties in Sweden (Dalarna, Värmland, Västmanland, and Örebro) except for wolf-killed moose where data from the counties of Gävleborg in Sweden and Hedmark in Norway also were used. We also used published data for some parameters (see Table 1. for details of parameters used in the calculations and data sources).

**Table 1:** Variable inputs used to estimate available biomass (kg/month) from moose killed by wolves, hunter harvest, vehicle collisions, and natural death within an area the size of an average annual wolf territory (900 km^2^) in Scandinavia with and without presence of wolves.

Letter	Parameter	Source
*k*	wolf-killed moose (number/month)	[20], [28]
*j*	age class of moose (calf or adult)	[19], [20]
*n_j_*	proportion of moose in category *j* in wolf kills	[19], [20]
*w_j_*	live weight of moose in category *j* (kg)	[20], [33]
*e*	proportion of edible biomass	[34]
*d*	wolf consumption (kg/wolf/day)	[35]
*o*	harvested moose (number/month)	this study
*s_j_*	proportion of harvested moose within wolf territories in category *j*	this study
*f*	proportion of live weight constituted of internal organs	unpublished data
*r*	moose killed in vehicle collisions on roads (number/month)	this study
*y*	proportion of vehicle-killed moose where the entire carcass was available for scavengers	this study
*m_j_*	proportion of moose in category *j* during winter	[28]
*p*	moose killed in collision on railways/km railway (number/month)	[38]
*h*	averaged distance of railway (km)	this study
*l_j_*	moose dying of natural causes in category *j* (number/month)	[39], [40]
*q*	weight loss of moose during hard winters (kg)	[42]
*z_j_*	wolf-killed moose in category *j* that is compensatory mortality (number/month)	[43]
*t_j_*	proportion of harvested moose in areas without wolves in category *j*	this study

#### Wolf-killed moose

The average number of moose killed by wolves per territory (***k***) in different age classes (***j***) during the summer period (1 June to 30 September) was calculated based on an increasing day interval between moose kills = (0.0068× day from 1 June +1.009)^2^ [20]. For winter (from 1 October to 31 May) we applied an average day interval between moose kills = 4.065 per territory [28]. Kill rates by wolves on moose were independent of pack size all year round [20], [28]. The mean annual number of moose killed by wolves was estimated based on the averaged values measured during different moose densities (median 1.4, IQR 1.0–1.5 moose/km^2^ [28]). The proportion (***n***) of calves (0–12 months old) and adults (>12 months old) in wolf kills was 90% and 10% respectively during summer [20], and 70% and 30% respectively during winter [19]. Body weight (***w***) of calves was calculated assuming linear growth [20] during summer starting with 13 kg as the live weight on 1 June and ending with 150 kg on 30 September. Throughout the winter we used a body weight of 150 kg for calves. For adults we used 300 kg all year round (as an estimate of the average weight of yearlings and adult males and females for this population [33], Sand et al. unpublished data).

Consumption by wolves and scavengers on wolf-killed moose were obtained from GPS-collared wolves in 17 territories (2001–2010) following methods described in [19] and [20]. In the calculation of estimated available biomass we used only wolf-killed and probably wolf-killed moose that were detected a maximum of four days after assumed time of death (the time of the first wolf GPS-location within 200 meters from the moose carcass [20]). The edible proportion of moose carcasses (***e***) was set to 65% of the total body weight [34]. The proportion of edible biomass consumed at the time of prey detection (when collared wolves were >2 km from the kill site) was visually estimated to the nearest 5%. We calculated the average proportion of biomass consumed during summer and winter for each age class of moose. To account for the consumption of the carcass by scavengers prior to time of carcass detection by field personnel we calculated wolf consumption (***d***) using the minimum daily food requirements of wolves (3.25 kg/wolf/day [35], wolf pack size (counted during winter), and days since prey detection (as a proxy for wolf handling time)). We did not account for consumption by wolves that revisited wolf-killed prey remains because the camera monitoring conducted in this study showed that revisits by wolves were short and rare (see Results). Nor did we account for losses to invertebrates. Consequently, the estimated biomass from wolf-killed moose (***b_w_***) was:
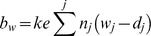



#### Hunter harvest of moose

We used data on the average number of harvested moose (***o***) in 61 management units inside 11 wolf territories during five consecutive years (in the period 2000–2008) obtained from the County Administrative Boards to estimate scavenger consumption of hunter-killed prey. The number of harvested moose was 0.4/km^2^±0.02 (mean±95% CI, n = 305), where calves and adults constituted 40% and 60% respectively (***s***, Table 2). Biomass from internal organs (***f***, (lungs, spleen, stomachs, intestines and sexual organs, and often also heart, liver and kidneys)) left behind by hunters was set at 17% of live weight based on the gut weights (rumen excluded) from calves (n = 91) and adults (n = 69, Sand et al. unpublished data) and assuming that the rumen constituted half of the weight. Dates of moose harvested (n = 41 063) were obtained from the Swedish Association for Hunting and Wildlife Management during four consecutive years (2007–2010). Moose were harvested in September (7%), October (75%), November (12%), December (4%), and January (2%). The amount of biomass available from hunter-harvest remains (***b_h_***) then was:
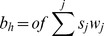



**Table 2:** Number of moose killed annually from different causes within an area the size of an average annual wolf territory (900 km^2^) with and without presence of wolves in Scandinavia.

Cause of death	Wolf present		Wolf absent	
	Calf	Adult	Calf	Adult
Wolf-killed	102	24	–	–
*additive*	*98*	*23*	–	–
*compensatory*	*4*	*1*	–	–
Hunter harvest	144	216	192	289
Vehicle collisions	5	11	5	11
*roads*	*4*	*9*	*4*	*9*
*railways*	*1*	*2*	*1*	*2*
Natural causes	10	10	14	11

#### Moose killed in vehicle collisions

We used data from the National Wildlife Accident Council [36] on the number of police-reported moose-vehicle collisions on roads and railways per month during six years (2003–2008, n = 5 609) to estimate the average annual number of moose in collisions on roads within a wolf territory. Approximately 80% of the moose hit by vehicles died (***r*** [37]) either immediately or assumed to have been shot later by search patrols. We used data of moose hit by vehicles and checked by search patrols (available between January 2010 and May 2011, n = 1 169) from the Swedish Association for Hunting and Wildlife Management to estimate the proportion of vehicle-killed moose that were retrieved for human consumption and where only internal organs were left for scavengers (∼60%). For the remainder (40%) we assumed that the entire carcass was left for scavengers (***y***). We also assumed that 15% of the moose involved in vehicle collisions survived, while the remaining 5% died but were never found (Seiler A, PhD, researcher in traffic mortality in wildlife, personal communication) and thus were available to scavengers. However, only ∼70% of all moose-vehicle collisions are estimated to be reported to the police (Seiler A, personal communication). Therefore, we adjusted for this bias from non-reported collisions but assumed that only 10% of the moose died because these accidents are unlikely to be as serious as those reported to the police (Seiler A, personal communication). The entire biomass from these carcasses were assumed available for scavengers. We also assumed the same age-class distribution of moose killed by vehicle collisions as found in the winter population (***m***, calves 0.3, adults 0.7 [28]).

Approximately eight moose were killed annually per 100 km railway (***p***, 15% each in January and February, 9% in March and in each month from September to December, and 5% in each month from April to August [38]). The average railroad density (***h***) in the study area was 0.045 km/km^2^. The majority of collisions on railways are directly lethal for the moose, and practically no moose hit by trains are retrieved for human consumption (Seiler A, personal communication). Therefore, we assumed that the entire biomass from train-killed moose was available for scavengers. The following formula was used to estimate biomass from vehicle collisions on roads and railways (***b_c_***):
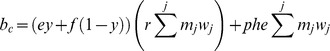



#### Moose dying of natural causes

The number of adult moose dying of natural causes (***l***, here defined as mortality not caused by human harvest, vehicle collisions, or predation) is approximately of the same magnitude as mortality from collisions with vehicles in areas without large predators (fraction of deaths of adults: vehicle 0.06, natural 0.08 [39]; vehicle 0.09, natural 0.10 [40]). Therefore, we assumed that 11 adult moose died of natural causes annually within a wolf territory, which is the corresponding number found for vehicle collisions (Table 2). Annual mortality from vehicle collisions and natural deaths combined was 0.05 for adult moose (1–13 years old) in our study area before wolf establishment [41]. This corresponded to 440 adult moose in an average wolf territory (22/0.05 = 440). The assumed proportion of 0.7 adults in the population gave the number of 189 calves in an average wolf territory (440/0.7–440 = 189). For calves, the annual mortality from vehicle collisions and natural causes combined was 0.10 [41], which gave the total number of calves dying from these two mortality causes (∼19). Subtracting the number of calves that died in vehicle collisions (5 according to the calculation above, Table 2) gave the number of calves dying of natural causes (14). We assumed a similar weight loss for all moose dying of natural causes all year round as found for calves during hard winters (***q***, 13% [42]). All natural mortality of calves occurred during winter [41], and we assumed a uniform distribution from January to April. For adults we assumed that 60% of the mortality occurred from January to April and 5% each month from May to December. Finally, we assumed 19% of wolf-killed moose calves (∼4) and 7% of adults (∼1) were compensatory (***z***) to natural mortality from January to April [43] and the corresponding biomass was reduced from the total amount of biomass from moose dying of natural causes. No data were available on compensatory mortality due to wolf predation during the rest of the year and we assumed it to be negligible. The estimated biomass from natural mortality (***b_n_***) was:
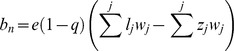



#### Scavenging in areas with and without wolves

The estimated available biomass for scavengers within wolf territories (***b_p_***) was calculated simply as: 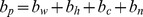


Hunters within a wolf territory must reduce the annual harvest if their purpose is to maintain a constant population density in the moose population [44]. Hunter harvest declined immediately after wolf re-colonization in Sweden [45]; therefore, we assumed that in areas without wolves the harvest was maintained at a level equal to the annual number of wolf-killed moose while accounting for compensatory mortality (natural death). We used data from harvested moose in 62 management units outside wolf territories during five consecutive years (in the period 2000–2008) obtained from the County Administrative Boards to estimate age-class distribution for harvested moose in areas without wolves (***t***, calves 40%, adults 60%, Table 2). Finally, we assumed that the rate of mortality from vehicle collisions on roads and railways was similar in areas with and without wolf presence (Table 2). Available estimated biomass for scavengers in areas without wolves (**b_a_**) was calculated as:
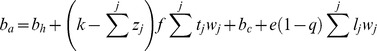



### Camera Monitoring

To monitor use of carcasses by scavenging species we placed movement-triggered cameras at carcasses of moose killed by wolves all year round (2006–2010) and at remains from hunter harvest in autumn (2007–2009). Cameras were set up when collared wolves where >2 km away from the carcass, and in three cases in territories without collared wolves. Sites with remains from moose harvest were reported by hunters and cameras were set up the same day as the moose were shot.

We used the cameras STC-WD1, STC-IR1 and STC-WD2-IR manufactured by Stealth Cam (Grand prairie, Texas, USA). Because red fox reacted to flash light used in camera brand STC-WD1, we removed the light and used only this type of camera during summer. Also, the light emitters were switched from camera brand STC-WD2-IR to emitters with longer wavelength creating invisible infrared light (LOKE Special Electronics, Skinnskatteberg, Sweden). Cameras were programmed to shoot three photos when triggered by movement with a minimum of one minute between triggering events. Date and time were registered on each photo. The majority of the data used in the analysis (85%) was from camera brand STC-WD2-IR.

Cameras were placed on tree stems approximately 0.5 m above ground and two to six meters away from carcasses. The proportion of edible biomass consumed was visually estimated at the time of camera set up and at each visit made by field personnel to replace battery and memory card (approximately once a month). Cameras were removed when carcasses were totally consumed or occasionally due to camera failure. The movement detectors were not triggered by birds smaller than jays or by mammals smaller than pine martens.

Presence of species and number of visits per species were determined from one of the three photos shot within each one-minute interval by choosing the one with the highest number of individuals observed. We pooled the number of camera days monitored per carcass into ten-day periods [46] and calculated the number of visits by any scavenger as well as for primary scavenging species separately per ten-day period. Species that constituted >5% of all visits at wolf kills were considered as primary scavengers. The ten-day periods were classified according to the season of the year (winter (January to March), spring (April to June), summer (July to September), and autumn (October to December)), consumption (three stages for wolf-killed moose), days since death of moose (according to wolf GPS-locations or an estimation), and wolf presence (i.e. visits by wolves at any point within the ten-day period). We used similar classes of consumption stage to [21]: stage 1: organs and/or major muscle groups (0–85% consumed), stage 2: minor muscle groups of bone and hide (90–95% consumed), or stage 3: only hide and bones (100% consumed). Classification was based on visual estimation of consumed parts in the photographs. Days since the death of the moose were classified into 24 ten-day periods (where the first time period included ten-day periods with camera set up between 0–9.9 days since the death of the moose) and used to investigate whether utilization of carcasses by scavengers differed with time.

### Analyses

#### Factors influencing carcass use by scavengers

We used generalized linear mixed model (GLMM) to enable modelling of variables measured at multiple time scales with an unbalanced design using SPSS Statistics 21.0 (IBM SPSS Inc., Chicago, Illinois, USA). We analysed factors influencing (1) presence at a carcass and (2) number of visits by scavenging species at wolf kills. Because carcasses were at different consumption stages at the start of the study period, they were sampled an unequal number of times resulting in an unbalanced design. Presence or absence of any scavenging species (all species pooled), as well as primary scavenging species separately, during ten-day periods were analysed using a binary logistic regression with carcass ID as the random effect to account for repeated observations. We used backward elimination of non-significant variables using 0.10 as the probability for removal where consumption stage, season of the year, and presence of wolf were entered as fixed factors and time since death as a covariate. Because 60 of the ten-day periods (n = 321) were shorter than the stipulated ten days, we tested if this affected the presence of scavengers using recording time as a covariate. If a significant effect was shown, we removed those ten-day periods (n = 14) and re-ran the analysis. The random effect covariance type was set to unstructured. We used the odds ratio (e^ß^) to quantify the change in the probability of being present relative to the change in fixed factors and a one unit change in the covariate. Factors were considered as significant at the α-level <0.05. In a second analysis that included only observations when scavengers were present, we determined the factors influencing the number of visits within ten-day periods using Poisson regression and the same random and fixed factors, covariates and covariance structure as in the presence/absence analysis with a robust estimation of fixed factors and parameter estimates.

#### Use of hunter-harvest remains versus wolf kills

To investigate that the probability of being present and number of visits at a carcass differed due to cause of death, we analysed the first three ten-day periods entering carcass ID as a random factor, cause of death as a fixed factor and time since death as a covariate as well as their interaction. To control for geographical variation in population densities of scavengers we restricted the comparison between the remains from hunter harvest and wolf kills within two bordering wolf territories. We used only wolf-killed moose recorded during the hunting season (October to January in those territories).

## Results

### Consumption of Wolf-killed Moose

A total of 117 wolf-killed moose were found within four days (average 2.5 days year round) after the estimated time of death. Of all carcasses, 49% were at consumption stage 1, 23% at stage 2, and 28% at stage 3 at the time of detection. The visually estimated proportion of edible biomass consumed was 70%±15 (n = 117, mean±95% CI) with an average of 80%±10 for calves (n = 50) and 55%±25 for adult moose (n = 9) during summer. The corresponding numbers during winter were 70%±10 for calves (n = 39) and 50%±15 for adults (n = 19). Of the total consumption, scavengers accounted for 6% of calves and 61% for adults during summer. The corresponding numbers during winter was 43% for calves and 45% for adults.

Average wolf pack size during winter was four (range 2–9, n = 26). The proportion of total edible biomass consumed (arcsine transformed) increased with increasing winter pack size when we accounted for age of prey (calf or adult), season (summer or winter) and time since death (0–4 days) (GLMM, B_1, 115_ = 0.039, SE = 0.019, t = 2.003, P = 0.048).

### Available Biomass for Scavengers

**Figure 1:**
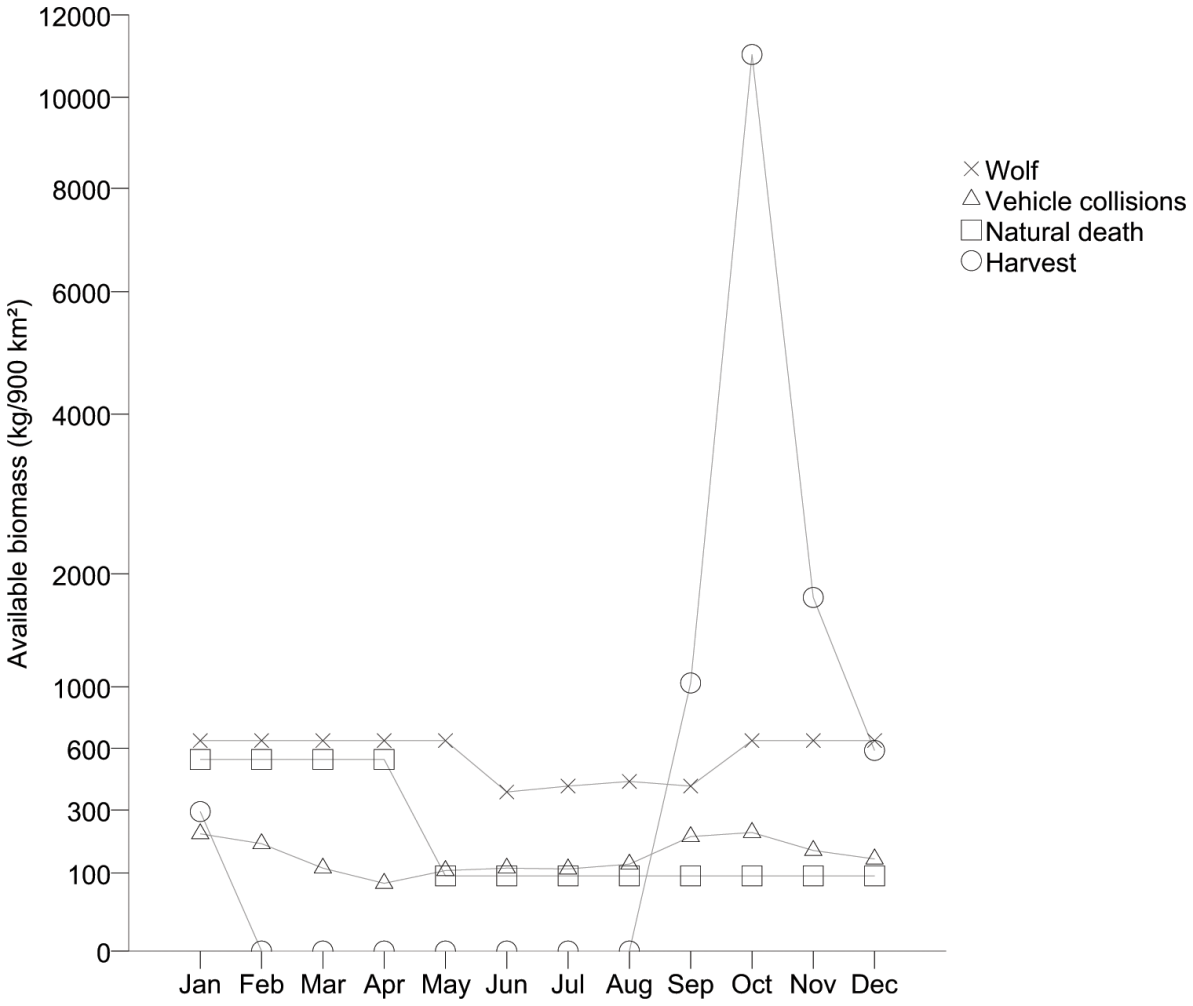
Estimated amount of biomass available for scavenging species from moose carcasses. Estimations of available biomass from wolf kills, hunter harvest, vehicle collisions, and natural death are conducted for an area corresponding to an average wolf territory (900 km^2^) in Scandinavia.

**Figure 2:**
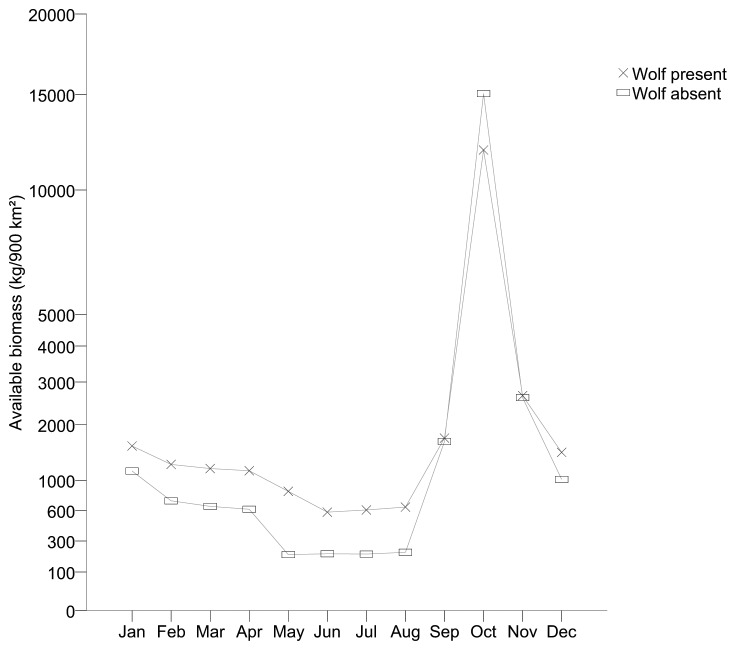
Estimated amount of biomass available for scavenging species in areas with and without wolves. Estimations of available biomass include wolf kills (only for areas with wolves present), hunter harvest, vehicle collisions, and natural death. Calculations are conducted for areas of 900 km^2^ corresponding to an average wolf territory in Scandinavia.

The greatest amount of estimated available biomass for scavengers during the year occurred during October due to the peak of moose hunting in that month in areas both with wolves (Figure 1) and without wolves (Figure 2). In areas with wolves present, wolf-killed moose contributed with 26% (per 900 km^2^ and year), hunter harvest with 57%, collisions with vehicles with 7%, and natural death with 10% of the total amount of biomass (Figure 1). In areas without wolf predation hunter harvest contributed with 81%, vehicle collisions with 7% and natural death with 12% of the biomass.

Presence of wolves resulted in a 6% higher estimate of the total annual amount of biomass available for scavengers (25 648 kg as compared to 24 289 kg without wolves present). The relative biomass estimated available to scavengers in wolf territories compared to areas without wolves ranged from 38–324% higher per month (481 kg more in wolf territories on average per month, range 377–644 kg) from December to August (Figure 2). In contrast, available relative biomass was estimated to be 20% (3 083 kg) lower in wolf territories during October and was estimated to be similar among areas with and without wolves during September and November (2–4%, only 48–66 kg difference).

### Carcass Use

We monitored 49 wolf-killed moose with movement-triggered cameras in 10 territories during 2 916 days (range 5–199 per carcass). The median day for the start of monitoring was four days (range 1–44) after moose death. The proportion of edible biomass consumed at the time of camera set up was 70%±10 (mean±95% CI). Of all moose carcasses, 53% were at consumption stage 1, 37% at stage 2, and 10% at stage 3. At 13 of the 49 wolf-killed moose sites, monitoring was not continuous due to malfunction of the camera or battery depletion.

**Figure 3:**
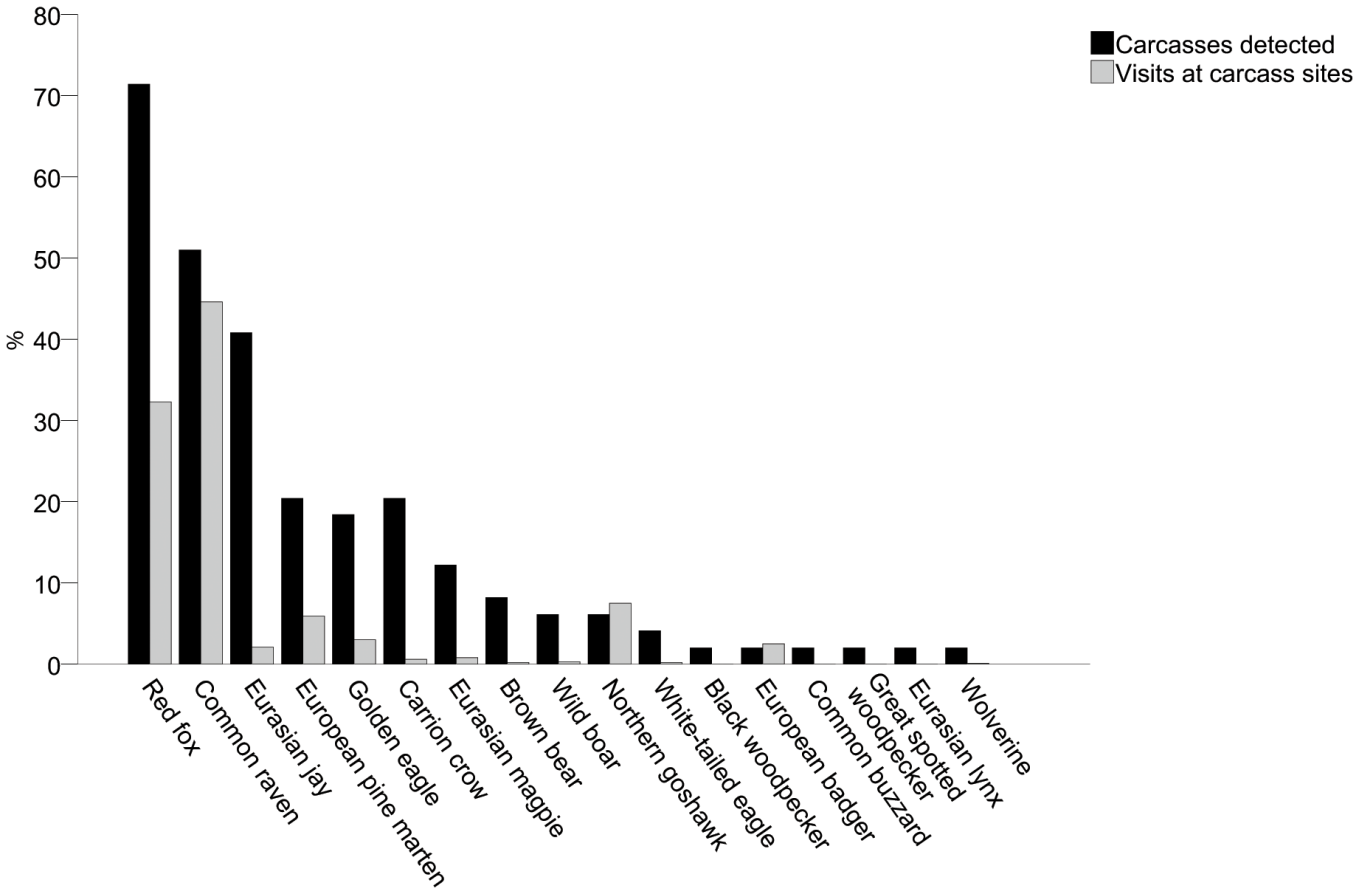
Utilization pattern by scavengers at wolf-killed moose. Proportion of wolf-killed moose (n = 49) detected by different scavenging species and proportion of visits to all wolf kills (n = 14 783) of respective species. Scavengers were recorded by movement-triggered cameras in Scandinavia.

A total of 13 055 photos were taken of visitors at the moose carcasses, including 14 783 visits by scavenging species (1–8 individuals per photo), 925 by wolves (at 11 carcasses), 101 by unidentified species, and 34 by species not classified as scavengers (ungulates, grain-eating bird species, hunting dogs, and humans). Another 3 397 photos contained no visitor (possibly triggered by wind, sunlight, or scavenging species inside movement detector range but outside camera range) and an additional 460 photos failed due to snow covering the lens or malfunction of the infrared light during night time. In total, 17 scavenging species (Figure 3) were registered at wolf-killed moose sites. Red foxes (n = 4 777), ravens (n = 6 588), pine martens (n = 868), and northern goshawks (*Accipiter gentilis*, n = 1 112) were the primary scavenging species and made up 90% of all visits by scavenging species. Revisits by wolves were short and rare (median 4 visits/ten-day periods, range 1–839 with 96% of visits at one carcass) where 63% occurred during consumption stage 2 and 3. Five out of the 11 carcasses that were revisited by wolves were adult moose but this did not differ from the proportion of adults among total wolf kills (2 out of 11, χ^2^ = 1.886, df = 1, P = 0.169).

The presence of any scavenger being at a wolf kill was influenced by the consumption stage with the highest probability in stage 1 and 2 compared to stage 3 (Table 3). This pattern was also evident for the red fox, raven, and pine marten individually. The season of the year and the presence of wolves were significant predictors of the presence of red foxes and ravens at a wolf kill site with wolf presence increasing the probability of their use and their presence being most likely during spring and summer, respectively (Table 3). The presence of any scavenger and red foxes individually significantly increased with increasing time since death of the moose. In contrast, the presence of pine martens at wolf-killed carcasses tended to decrease with time since death (Table 3). It was not possible to analyse the presence of goshawks at wolf kills because during only 15 out of the 321 time periods were wolf kills visited.

**Table 3:** Parameter estimates (ß) of consumption stage, season of the year, presence of wolves, and time (10-day interval) since death of moose on presence/absence by scavenging species to wolf-killed moose (n = 49) during ten-day periods (n = 321).

Species	Variables	ß	SE	P	Odds ratio	95% CI for odds ratio	
						Lower	Upper
Any scavenger	Consumption stage 1	1.873	0.418	<0.001	6.506	2.860	14.797
	Consumption stage 2	1.586	0.390	<0.001	4.882	2.267	10.511
	Consumption stage 3	0	0				
	Time since death	0.138	0.072	0.058	1.148	0.995	1.324
Red fox	Consumption stage 1	1.295	0.419	0.002	3.652	1.600	8.335
	Consumption stage 2	0.997	0.374	0.008	2.710	1.298	5.658
	Consumption stage 3	0	0				
	Winter	1.043	0.617	0.092	2.837	0.842	9.561
	Spring	1.638	0.623	0.009	5.145	1.510	17.526
	Summer	0.899	0.809	0.267	2.457	0.500	12.071
	Autumn	0	0				
	Wolf absent	−1.771	0.709	0.013	0.170	0.042	0.687
	Wolf present	0	0				
	Time since death	0.125	0.056	0.025	1.133	1.016	1.264
Common raven	Consumption stage 1	2.126	0.496	<0.001	8.378	3.157	22.237
	Consumption stage 2	1.620	0.451	<0.001	5.053	2.081	12.272
	Consumption stage 3	0	0				
	Winter	0.068	0.732	0.926	1.071	0.254	4.521
	Spring	1.310	0.726	0.072	3.705	0.888	15.453
	Summer	2.227	0.921	0.016	9.276	1.514	56.827
	Autumn	0	0				
	Wolf absent	−1.447	0.636	0.024	0.235	0.067	0.823
	Wolf present	0	0				
Pine marten	Consumption stage 1	4.351	1.247	0.001	77.523	6.673	900.641
	Consumption stage 2	2.634	1.129	0.020	13.929	1.511	128.364
	Consumption stage 3	0	0				
	Time since death	−0.404	0.220	0.067	0.668	0.433	1.028

Analyses were done for all species pooled as well as for the primary scavenging species separately (except goshawk) with backward elimination of non-significant variables.

Season of the year: winter (January to March), spring (April to June), summer (July to September), and autumn (October to December).

The number of visits to wolf kills was also influenced by the consumption stage with the highest use in stage 1 and 2 compared to stage 3 for any scavenger, and red fox, pine marten, and goshawk individually (Table 4). Pine martens and goshawks had the highest visitation rate at wolf kills during winter while visitation rates by red fox and ravens were highest during spring (Table 4). The number of visits to wolf kills increased with presence of wolves for any scavenger, with a similar tendency for red foxes, but the opposite was shown for pine martens (Table 4). Goshawks did not visit wolf kills during spring and summer, nor did they when wolves were present. The number of visits decreased with increasing time since death of moose for any scavenger and for ravens individually whereas this was not the case for red foxes, pine martens or goshawks (Table 4). Visits of raven constituted 45% of the total number of visits for any scavenger and had therefore a strong effect on the pooled data in this case.

**Table 4:** Parameter estimates (ß) of consumption stage, season of the year, presence of wolves, and time since death of moose on number of visits by scavenging species to wolf-killed moose (n = 49), during ten-day periods (n = 223).

Species	Variables	ß	SE	P	Odds ratio	95% CI for odds ratio	
						Lower	Upper
Any scavenger	Consumption stage 1	1.796	0.307	<0.001	6.025	3.293	11.026
	Consumption stage 2	0.989	0.422	0.020	2.688	1.171	6.173
	Consumption stage 3	0	0				
	Winter	0.744	0.378	0.050	2.105	0.999	4.435
	Spring	2.209	0.473	<0.001	9.102	3.584	23.120
	Summer	1.334	0.865	0.124	3.797	0.690	20.876
	Autumn	0	0				
	Wolf absent	−0.316	0.140	0.025	0.729	0.554	0.960
	Wolf present	0	0				
	Time since death	−1.291	0.259	<0.001	0.275	0.165	0.459
Red fox	Consumption stage 1	3.288	0.660	<0.001	26.783	7.273	98.631
	Consumption stage 2	2.320	0.745	0.002	10.171	2.332	44.360
	Consumption stage 3	0	0				
	Winter	0.810	0.692	0.244	2.247	0.572	8.825
	Spring	1.798	0.685	0.010	6.037	1.561	23.352
	Summer	−1.279	1.009	0.207	0.278	0.038	2.044
	Autumn	0	0				
	Wolf absent	−0.314	0.182	0.086	0.731	0.510	1.046
	Wolf present	0	0				
Common raven	Winter	1.455	0.400	0.001	4.283	1.929	9.512
	Spring	2.418	0.213	<0.001	11.223	7.342	17.156
	Summer	0.590	0.508	0.250	1.804	0.654	4.977
	Autumn	0	0				
	Time since death	−1.520	0.336	<0.001	0.219	0.112	0.428
Pine marten	Consumption stage 1	2.089	1.396	0.146	8.079	0.461	141.662
	Consumption stage 2	1.822	0.694	0.014	6.182	1.487	25.702
	Consumption stage 3	0	0				
	Winter	2.442	0.569	<0.001	11.491	3.573	36.958
	Spring	1.771	0.608	0.007	5.878	1.687	20.483
	Summer	no visits	no visits				
	Autumn	0	0				
	Wolf absent	1.057	0.332	0.004	2.877	1.454	5.690
	Wolf present	0	0				
Goshawk	Consumption stage 1	5.671	0.094	<0.001	290.422	236.233	357.041
	Consumption stage 2	3.756	0.038	<0.001	42.797	39.395	46.494
	Consumption stage 3	0	0				
	Winter	1.428	0.016	<0.001	4.169	4.027	4.317
	Spring	no visits	no visits				
	Summer	no visits	no visits				
	Autumn	0	0				

Analyses were done for all species pooled as well as for the primary scavenging species separately, with backward elimination of non-significant variables.

Season of the year: winter (January to March), spring (April to June), summer (July to September), and autumn (October to December).

#### Wolf kills versus remains from hunter harvest

For this analysis we used a subset of 11 wolf-killed moose during 32 ten-day periods and 11 remains from hunter harvest during 31 ten-day periods. The proportion of edible biomass consumed at wolf kills was 65%±10 (mean±95% CI). We registered ten scavenging species at wolf-killed moose in this subset and nine at remains from hunter harvest. The same scavengers visited carcasses except wild boar (*Sus scrofa*) and wolverine scavenged only wolf kills and the Eurasian eagle-owl (*Bubo bubo*) scavenged only hunter-harvest remains. There was a total of 1 519 visits by scavengers to the wolf kills compared to 493 visits to the hunter-harvest remains, of which 260 and 220 visits were made during the first ten-day period, respectively. Ravens (n = 56), pine martens (n = 251), jays (n = 41), magpies (*Pica pica*, n = 73), and golden eagles (*Aquila chrysaetos*, n = 53) were the primary scavenging species at remains after hunter harvest and made up 96% of all visits by scavenging species. Primary scavengers at this subsample of wolf kills were ravens (n = 343), pine martens (n = 183), jays (n = 152), and goshawk (n = 646) and made up 87% of all visits.

Carcass sites with remains after hunter harvest tended to have a higher probability of being visited at all but visited fewer times than wolf kills (Table 5). Presence at carcass sites did not change with time since moose death, whereas the number of visits decreased (Table 5). The presence of scavengers decreased faster with time at remains from hunter harvest compared to wolf-killed moose, but this pattern was not evident for the number of visits at carcass sites (Table 5). There was no clear indication that the primary scavenging species of wolf kills (n = 49) visited the two types of carcasses differently with the exception of goshawks who showed a lower presence at remains from hunter harvest. Red foxes were present at wolf-killed moose in 16% of the time periods and 13% at remains from hunter harvest. The corresponding numbers for ravens, pine martens and goshawks were 13% and 19%, 28% and 23%, and 16% and 3%. We never recorded wolves at remains after hunter harvest.

**Table 5:** Parameter estimates (ß) of cause of death (hunter harvest (n = 11) or wolf kill (n = 11)) and time since death (ten-day periods (n = 63)) and their interactions on presence/absence and number of visits by any scavenging species (all species pooled).

Model	Variables	ß	SE	P	Odds ratio	95% CI for odds ratio	
						Lower	Upper
Presence/absence	Hunter harvest	2.320	1.217	0.061	10.171	0.891	116.058
	Wolf kill	0	0				
	Time since death	0.215	0.691	0.757	1.240	0.311	4.940
	Hunter harvest × Time since death	−2.713	1.211	0.029	0.066	0.006	0.749
	Wolf kill × Time since death	0	0				
Number of visits	Hunter harvest	−1.028	0.589	0.090	0.358	0.108	1.184
	Wolf kill	0	0				
	Time since death	−0.819	0.266	0.004	0.441	0.257	0.757
	Hunter harvest × Time since death	−0.197	0.513	0.704	0.821	0.290	2.331
	Wolf kill × Time since death	0	0				

## Discussion

### Provision of Moose Carrion in Scandinavia

The re-colonization of Scandinavia by wolves has only marginally increased the total annual amount of estimated biomass available for scavengers, although wolf-kills contributed up to one fourth of the annual estimated moose carcass biomass within wolf territories. Wolf predation is partly compensatory to other sources of moose mortality (natural death [43] and hunter harvest [45]), reducing their respective contributions of carrion. However, as also demonstrated in Greater Yellowstone [5], the most important effect of wolves to the scavenger community in Scandinavia was rather the reduction of the high seasonal variation of available moose carrion. Wolves reduced the peak of carrion biomass during the autumn hunt, and increased the amount of carrion during the rest of the year. The highest increase occurred from May to August when wolf predation was considered additive to other natural moose mortality, while from January to April when the predation was partly compensatory to natural mortality [43], the increase was less.

In this study we focused on modelling the average impact of wolves on the temporal availability of biomass to scavengers in order to compare the importance of wolf predation with other causes of moose mortality. In our comparison between areas with and without wolves, we have not accounted for the difference in number of vehicle killed moose due to the lower density of roads inside wolf territories compared to areas without wolves [47] as it is known that road density will affect the number of collisions [6]. However, vehicle kills only constituted 7% of all biomass available to scavengers and a reduction of this within wolf territories is not likely to change the seasonal pattern of carrion availability. It is likely that natural mortality varies more within and between years than wolf predation, hunter harvest, and vehicle collisions, although detailed data on the availability of carcasses over time is needed to substantiate this. Both density-dependent resource limitations during winter and density-independent factors, like weather conditions that influence food quality, are important factors affecting mortality year round resulting in a variation in natural mortality among ungulates, areas, and years [48]. However, as there is hardly any difference in biomass from natural mortality between areas with and without wolves (due to low compensatory mortality), it is unlikely that our conclusion on wolves altering biomass flow is violated.

Food provisioning to scavengers depends both on social system and group size in large predators; e.g. solitary pumas (*Puma concolor*) provides a greater amount of biomass compared to large wolf packs [49]. Comparing Scandinavian wolves to puma in South America reveal a four times higher provisioning of biomass by pumas. After the reintroduction of wolves in Yellowstone, the availability of carcasses was dependent on both kill rate and wolf pack size where intermediate pack sizes provided the largest biomass for scavengers [21]. In Scandinavia, the wolf kill rate of moose is independent of pack size [20], [28], and the remains of carcasses left for scavengers will therefore decrease with increasing wolf pack size. However, not all wolf pack members feed on all carcasses at the same time, as the entire pack does not always travel together [31] and pack cohesion varies with season and pack size [50] resulting in an intra-territory variation in available biomass from wolf kills. Wolf kills are likely less spatially aggregated than carcass remains after hunter harvest and vehicle collisions [5], [6] which may benefit scavengers with relatively short feeding radii [5]. The amount of biomass available to scavengers in Scandinavia is also likely to show a spatial variation among wolf territories due to a substantial variation in wolf territory size (200–1 500 km^2^ [32]). This variation seems not to be correlated to variation in kill rates [32]. In addition, other factors like moose density, prey-to-predator ratio, and moose population structure (proportion of calves) may influence wolf kill rates [28]. Taken together this suggests that wolves will have a low impact on the production of carrion in some areas and a relatively large in others.

### Scavenging Patterns and Consequences for the Scavenging Guild

There are no obligate mammalian or avian scavengers in Scandinavia. All the species with at least the size of jay, that we expected to be facultative scavengers, were documented scavenging on wolf kills, although the majority of the species were only observed on few occasions. Similar to Białowieża Primeval Forest, Poland, which has a comparable guild of facultative scavengers as central Scandinavia [29], red fox, raven and jay were the dominating species present at carcasses. The first two were also the most frequent visitors to wolf kills. The low frequency of visits by jays, despite a high number of carcasses detected, may be a result of their small body size, which might have failed to trigger the movement detectors of the cameras at many visits. The use of carcasses by jays may therefore have been underestimated.

We found only minor differences in the number of species visiting wolf kills compared to remains from hunter harvest, but wolf kills tended to have a higher number of visits than remains from hunter harvest, even during peak harvest month (October). The probability of scavengers being present decreased faster with time at remains from hunter harvest than at wolf kills, likely a result of less biomass available per carcass. This is further supported by wolf-killed moose, with the highest amount of meat (consumption stage 1), also had the highest probability of visitation and greatest number of visits by scavengers. Remains from harvested moose may provide higher nutritional quality [35] per unit weight than wolf kills and therefore to some extent offset the smaller quantity of carrion biomass per single dead moose. For example, internal organs are vital sources of essential fatty acids, which may be the reason why wolves usually start their consumption with these parts of newly killed ungulate prey [35].

Strong positive effects of carrion on the population dynamics of scavengers are generally assumed in wildlife ecology literature, although there is a paucity of such data [1], [21], [29], [51]. Our study revealed that the presence of wolves creates a more even distribution of carrion biomass over the year. This pattern is likely to be especially important during spring when scavenging species breed and provide for dependent growing offspring, and therefore have a higher energy demand. This is further supported by both the percentage of wolf kills visited and the number of visits by scavenging species to wolf-killed moose was highest during spring. Spring is also the season when wolves increased the available carrion biomass by two to four times compared to areas without wolves. Actually the provision of moose carrion during spring was even higher, as these figures are based on the number of moose killed by wolves during this period. In contrast to warmer regions where carcasses are decompose in relatively a short time [46], carcasses in northern colder ecosystems last longer. This is especially true for large animals like moose killed during winter. Such carcasses were often more accessible during spring when snow melt made them visible and rising temperatures made them easier to handle for scavengers. The exceptions were pine marten and goshawk that mainly utilized carcasses during late winter. At cold temperatures, pine marten reduce their activity and stay in well-insulated sites, close to carcasses where they can frequently feed [52]. Also the composition of the diet of goshawk change drastically between different seasons of the year [53].

In contrast to findings in more remote wilderness areas [15], wolves in our study area showed a low tendency to return to old kill sites, possibly because of high density and a pronounced vulnerability of their main prey species, making new kills relatively easy [20], [27]. Therefore, scavenging species will have access to a large part of the available biomass once wolves leave their kills. Competition between wolves and scavengers regarding carcasses with other causes of death is also likely minor as wolves did not scavenge remains from hunter harvest.

The return of wolves to the Scandinavian ecosystem may not be exclusively positive for the scavenging guild if it results in intra-guild predation by wolves. However, as wolves in our study area do not rest in the immediate vicinity of kill sites [19], the risk of scavengers encountering wolves at carcasses is low. This seem to be in contrast to the situation in North America where wolves commonly rest <100 meter from their kills increasing the risk for scavenging species to encounter wolves [35]. However, we did not find any evidence of wolf-killed scavengers at kill sites in our study. Although there was a low tendency of wolves returning to old kill sites, those sites where this happened were also those most frequently visited by red fox and raven, supporting previous studies showing that these scavengers follow wolves to find carcasses [11], [12].

As red fox and raven (and possibly also jay) were the most frequent visitors at wolf kills, these species may benefit the most from the return of the wolf. However, these species are also the most abundant members of the scavenging guild, and it is possible that their high frequency of visits can be only a function of their high abundance. Consequently, provision of carcasses by wolves may be as important or even more important for rarer species like the golden eagle (listed as a near threatened species [30]) or goshawk, if the low visit frequency is a consequence of low abundance rather than low use. An interesting species in this context is the wolverine, which recently has expanded from its former stronghold in the northern alpine areas in Scandinavia, south into the current wolf range in the forested areas of south-central Sweden [45]. Although we only had few visits by wolverines at kill sites, it is possible that wolf kills may promote colonization of wolverines into the south-central parts of Scandinavia [54] as wolverines are highly dependent on carcasses provided by other large predators [55]. In order to estimate the relative importance of carrion for different scavenging species, one would need to compare the frequency of visits in relation to quantitative data on their relative abundance, data that is currently lacking in this system.

### Conclusion

Our findings demonstrate that when wolves colonize an ecosystem that is intensely exploited by humans such as in Scandinavia, hunter harvest still provide the greatest amount of moose biomass to scavengers even if only for a few months in autumn. During the rest of the year, wolves play an important role in making biomass available to scavengers that also consume large parts of wolf kills. Although we lack quantitative data on how this may affect the demography of scavenging species, it is likely that this will have consequences for the population dynamics of several species within the scavenger guild as the highest utilization of wolf kills occurred during spring, a period critical to reproduction and survival of young.
